# IMPACT OF AN EDUCATIONAL INTERVENTION ON KNOWLEDGE AND ATTITUDES ABOUT MEMORY LOSS AND CARE ISSUES

**DOI:** 10.1093/geroni/igac059.1269

**Published:** 2022-12-20

**Authors:** Colleen Galambos, Julie Ellis

**Affiliations:** University of Wisconsin-Milwaukee, Milwaukee, Wisconsin, United States; University of Wisconsin Milwaukee, Milwaukee, Wisconsin, United States

## Abstract

While previous studies have evaluated the impact of education interventions on knowledge and attitudes about Alzheimer’s disease and other dementias (Kaf, Barboa, Fisher, & Snavely, 2011; Kimzey, Mastel-Smith, & Alfred, 2016; Yamashita, Kinney, & Lokon, 2011), there is little research about the impact on knowledge and attitudes about issues related to caregivers of those with dementia. This paper reports on the results of an education intervention to increase college students’ knowledge, and positive attitudes on memory loss, Alzheimer’s disease and other dementias, and related caregiver issues. The intervention consisted of a series of lectures and readings, including a caregiver memoir. The intervention was conducted in social work and nursing classes over two semesters. A questionnaire, using established scales that measured knowledge and attitudes about dementia and caregiving issues, was administered to the students using a pretest-posttest design. The educational intervention tested successfully increased knowledge and positive attitudes about Alzheimer’s disease, dementia, memory loss, and caregiving (p=.0001). This finding held across courses, grade levels, previous experience with older adults, knowledge of someone with memory loss, and relationships with caregivers and persons with memory loss. A memoir as a storytelling informational narrative, and structured lectures and readings served as effective learning tools that influenced both knowledge and attitude levels about Alzheimer’s disease, dementia, memory loss, and caregiving. Gains made in knowledge and attitude changes were true for both face- to- face and online teaching venues. Implications for training, classroom teaching, and workforce development will be discussed.

